# GCS-YOLOv8: A Lightweight Face Extractor to Assist Deepfake Detection

**DOI:** 10.3390/s24216781

**Published:** 2024-10-22

**Authors:** Ruifang Zhang, Bohan Deng, Xiaohui Cheng, Hong Zhao

**Affiliations:** 1 Key Laboratory of Advanced Manufacturing and Automation Technology, Education Department of Guangxi Zhuang Autonomous Region, Guilin University of Technology, Guilin 541006, China; 2120221175@glut.edu.cn (B.D.); zhaohong@glut.edu.cn (H.Z.); 2 College of Mechanical and Control Engineering, Guilin University of Technology, Guilin 541006, China; 3 College of Information Science and Engineering, Guilin University of Technology, Guilin 541006, China; cxiaohui@glut.edu.cn

**Keywords:** deepfake, face extraction, lightweight, YOLOv8, HGStem, C2f-GDConv, P6, CCN-based Cross-Scale Feature Fusion with GDConv, Group Normalization and Shared Convolution Detect Head

## Abstract

To address the issues of target feature blurring and increased false detections caused by high compression rates in deepfake videos, as well as the high computational resource requirements of existing face extractors, we propose a lightweight face extractor to assist deepfake detection, GCS-YOLOv8. Firstly, we employ the HGStem module for initial downsampling to address the issue of false detections of small non-face objects in deepfake videos, thereby improving detection accuracy. Secondly, we introduce the C2f-GDConv module to mitigate the low-FLOPs pitfall while reducing the model’s parameters, thereby lightening the network. Additionally, we add a new P6 large target detection layer to expand the receptive field and capture multi-scale features, solving the problem of detecting large-scale faces in low-compression deepfake videos. We also design a cross-scale feature fusion module called CCFG (CNN-based Cross-Scale Feature Fusion with GDConv), which integrates features from different scales to enhance the model’s adaptability to scale variations while reducing network parameters, addressing the high computational resource requirements of traditional face extractors. Furthermore, we improve the detection head by utilizing group normalization and shared convolution, simplifying the process of face detection while maintaining detection performance. The training dataset was also refined by removing low-accuracy and low-resolution labels, which reduced the false detection rate. Experimental results demonstrate that, compared to YOLOv8, this face extractor achieves the AP of 0.942, 0.927, and 0.812 on the WiderFace dataset’s Easy, Medium, and Hard subsets, representing improvements of 1.1%, 1.3%, and 3.7% respectively. The model’s parameters and FLOPs are only 1.68 MB and 3.5 G, reflecting reductions of 44.2% and 56.8%, making it more effective and lightweight in extracting faces from deepfake videos.

## 1. Introduction

Deepfake technology is a product of continuous advancements in deep learning. With the evolution of science and technology, AI-generated face-swapping videos created using deepfake techniques have become a focal point of public attention. This technology leverages advanced deep learning frameworks such as Autoencoders (AE) and Generative Adversarial Networks (GAN) to generate synthetic faces with the aim of replacing the original face in a real video, thereby altering its content [1,2]. While deepfake technology has greatly contributed to industries like filmmaking, artistic creation, and virtual reality, it also poses significant risks, particularly in terms of privacy invasion, the creation of fake news, harmful pornographic content, and fraudulent activities [3,4,5]. As a result, research on detecting fake visual content generated by deepfake technology has become a topic of widespread concern in the international community.

Detecting deepfake videos requires first detecting and extracting faces from the video, a task categorized under object detection in the field of computer vision [6]. Object detection algorithms are generally divided into two categories: two-stage algorithms and one-stage algorithms. Two-stage algorithms generate candidate regions using methods like Region Proposal Networks (RPN), followed by further feature extraction using Convolutional Neural Networks (CNNs) for classification and precise bounding box regression. While two-stage algorithms typically offer superior accuracy, their detection speed is often insufficient. Representative algorithms in this category include R-CNN [7], Faster R-CNN [8], and MTCNN [9]. R-CNN utilizes the selective search algorithm to generate candidate regions, achieving high detection accuracy. However, it performs convolutional computations on each candidate region, leading to very slow processing speed, making it unsuitable for real-time detection tasks, and it consumes significant memory. Faster R-CNN improves upon R-CNN by optimizing detection speed, but the model’s complexity increases, requiring substantial computational power. MTCNN is designed specifically for face keypoint detection and is relatively lightweight, but it has lower detection accuracy, particularly underperforming in large-scale or complex face scenarios.

Unlike two-stage algorithms, one-stage algorithms directly predict object classes and bounding boxes within the network, bypassing the region proposal step. This results in faster detection speeds, albeit at the cost of some accuracy. Representative algorithms in this category include YOLO [10], SSD [11], and RetinaNet [12]. YOLO is an end-to-end detection algorithm known for its fast detection speed and compact model size, although its accuracy is lower compared to Faster R-CNN. SSD employs multi-scale feature maps for detection, allowing it to effectively capture faces of various sizes, but its performance declines in densely populated scenes. RetinaNet introduces Focal Loss, which enhances its ability to address class imbalance, making it particularly suitable for detecting rare or small-scale objects. However, its network architecture is complex and computationally intensive.

The algorithms mentioned above exhibit high performance in face detection tasks. However, when detecting faces in deepfake videos, the high compression rate of these videos often leads to blurred target features, resulting in false positives on non-face areas. Additionally, existing face detection algorithms demand substantial computational resources, placing high requirements on the memory and processing power of the experimental equipment. To address these challenges, we present a lightweight face extractor to assist deepfake detection, GCS-YOLOv8, which effectively reduces model parameters and computational load while maintaining high detection accuracy, enabling the reliable extraction of faces from fake videos. The main contributions of this paper are as follows:

(1) In the Backbone section, we utilize HGStem to improve the original downsampling operation of the model, reducing false positives on small non-face objects. We also propose GDConv and integrate it with the C2f module to create the C2f-GDConv module, addressing the low-FLOPs pitfall and reducing model parameters. Additionally, we add a P6 large-object detection layer, expanding the model’s receptive field and enhancing its ability to learn multi-scale features. This allows for the accurate extraction of face information from highly compressed deepfake videos, improving performance.
(2) In the Neck section, we design the cross-scale feature fusion module, CCFG, which effectively integrates features from different scales, improving the model’s adaptability to scale variations, reducing false positives on non-face areas, and significantly lowering model complexity. Compared to traditional face detectors, this makes the model more lightweight.
(3) In the Head section, we propose a novel lightweight detection head called GSCD (Group Normalization and Shared Convolution Detect Head), which significantly improves accuracy in single-object detection tasks such as face detection, while effectively reducing the computational resources required by the model.
(4) We refine the training dataset by removing low-accuracy small face labels, which enhances detection accuracy.

## 2. Related Work

### 2.1. Face Detection

Face detection has long been a focal point of research. This section reviews the development of face detection by introducing detectors from two different stages of algorithms proposed in recent years.

Two-stage algorithms: Tang et al. [13] proposed a multi-scale face detection method called PyramidBox, which enhances the detection accuracy of small faces by incorporating contextual information. Zhang et al. [14] introduced RefineDet++, a one-stage detector that employs a two-stage strategy during regression to refine predictions and achieve higher detection accuracy. Liu et al. [15] developed the MogFace face detection algorithm, which selectively fuses multi-scale features using a gating mechanism to filter out irrelevant information and improve detection performance.

One-stage algorithms: Xu et al. [16] designed an anchor-free face detection method called CenterFace, which detects the center point of a target to regress the corresponding bounding box size and keypoints, thereby effectively saving computational resources. Guo et al. [17] proposed an efficient one-stage face detector, SCRFD, which enhances detection accuracy through refined strategies and a feature pyramid network, while its lightweight network architecture ensures faster inference speeds. Qi et al. [18] optimized YOLOv5 and introduced the YOLO5Face face detector, utilizing a StemBlock and smaller kernel in the SPP module to achieve higher detection accuracy.

### 2.2. YOLOv8

**Figure 1:**
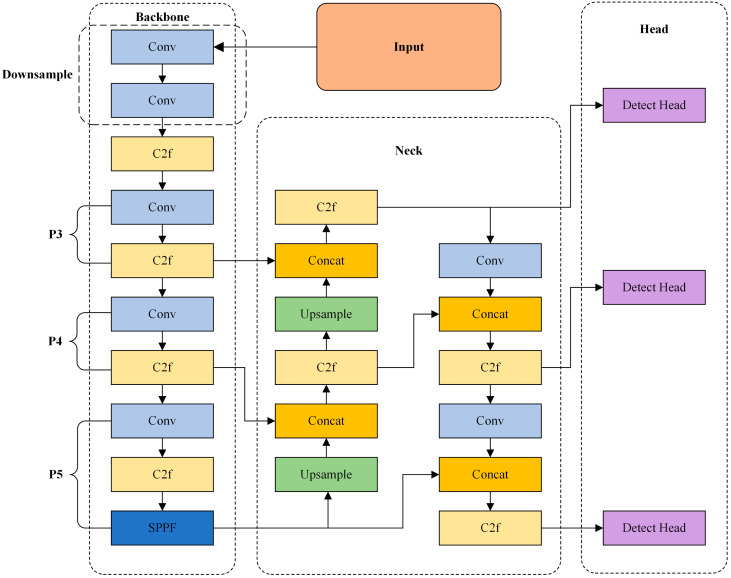
Structure of the YOLOv8.

YOLOv8 [19], developed by Ultralytics, is a novel object detection model that builds upon and improves the previous YOLO iterations, offering speed, accuracy, and ease of use. YOLOv8 consists of three main components: the Backbone, Neck, and Head. The Backbone, which serves as the primary feature extraction network, utilizes the CSPDarkNet architecture for feature extraction. It includes CBS (Conv + Batch Normalization + Sigmoid Liner Unit) modules and C2f modules, and employs the SPPF (Spatial Pyramid Pooling-Fast) module to enhance the network’s feature extraction capabilities. The Neck is a feature fusion network that incorporates the Path Aggregation Network–Feature Pyramid Network (PAN-FPN) approach, merging multi-scale features to strengthen the network’s feature representation. The Head is responsible for executing downstream tasks. In this paper’s task, it comprises a detection head and a classification head. The detection head generates detection results through regression loss, while the classification head categorizes each feature map. The network structure of YOLOv8 is illustrated in Figure 1.

## 3. Methods

**Figure 2:**
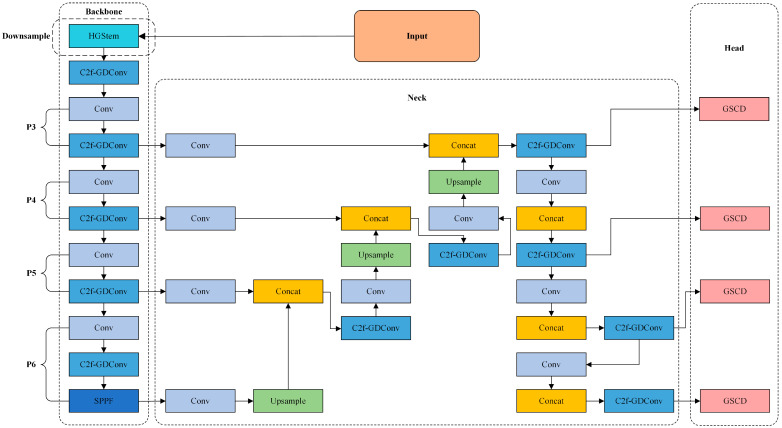
Structure of the GCS-YOLOv8.

To address the challenges of extracting faces from deepfake videos, we propose a novel lightweight face extractor specifically designed for deepfake videos. Based on YOLOv8, we replace the dual convolutional downsampling with HGStem, design the C2f-GDConv module to replace the C2f module, add a P6 large object detection layer, and develop the CCFG to improve the feature fusion network. Additionally, we introduce a lightweight detection head, GSCD. These improvements make the network more compact while enhancing model performance, effectively addressing the issues of false detections and missed detections in the face extraction process for deepfake videos. We name the modified YOLOv8 model GCS-YOLOv8, and its network structure is illustrated in Figure 2. The following sections will provide a module-by-module introduction of the improvements proposed in this paper.

### 3.1. Downsampling Module HGStem

**Figure 3:**
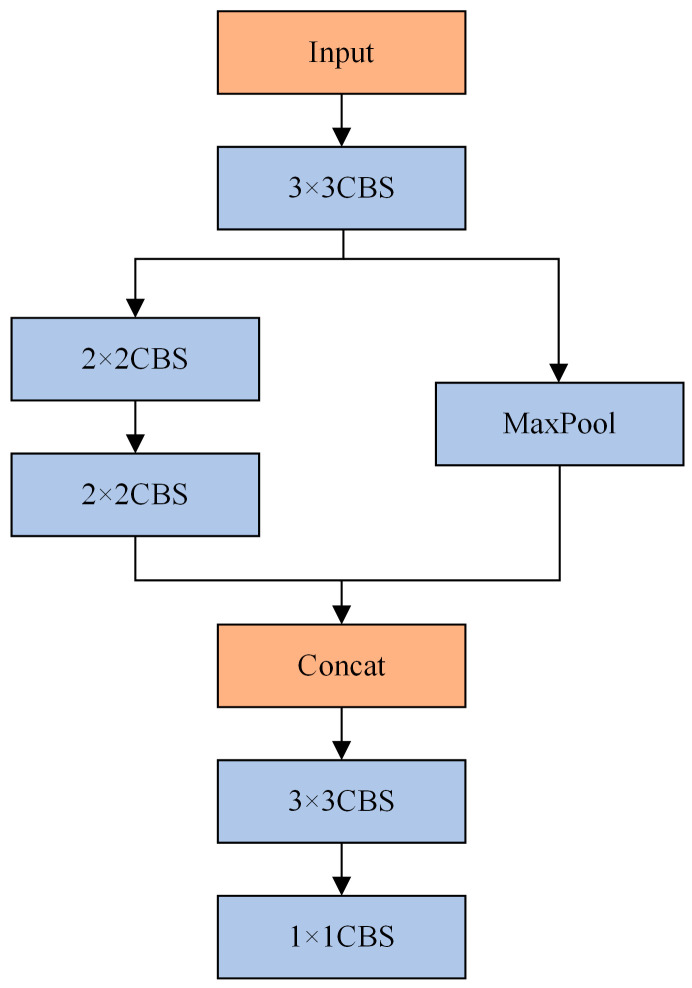
Structure of the HGStem.

YOLOv8 uses dual convolution for initial downsampling, achieving a 4x reduction in spatial dimensions. However, this approach can result in the loss of crucial feature information for small targets. In deepfake videos, besides the target face, there are often many small-scale non-face objects. If the correct feature information is not adequately learned, it can easily lead to false detections during the detection process. StemBlock was introduced in YOLOv5 to replace dual convolution for downsampling [18], which improved detection accuracy and demonstrated its effectiveness. Inspired by this, we innovatively introduce HGStem [20] to replace the dual convolution in YOLOv8 for initial downsampling. HGStem’s structure is similar to StemBlock, utilizing a branch structure that effectively reduces the model’s parameters and computational load. However, HGStem incorporates more CBS layers, enabling the network to extract features more quickly in the early stages. Compared to the original downsampling, HGStem can capture features at different scales, helping the network in learning various features. The structure of HGStem is illustrated in Figure 3.

HGStem takes the input feature map and first processes it through a 3 × 3 CBS module. The output is passed through a branching structure. One branch uses a depthwise convolution structure composed of two 2 × 2 CBS modules, which reduces computational load while extracting small-scale features. The other branch uses max pooling to retain the essential feature information of the feature map. The outputs of these two branches are concatenated, followed by further feature extraction and downsampling through another 3 × 3 CBS module, reducing the spatial dimensions of the feature map. Finally, a 1 × 1 CBS module adjusts the output channels. This module maximizes the retention of feature information while reducing parameters and computational load, effectively addressing the issue of false detections.

### 3.2. C2f-GDConv Module

In large-scale tasks such as face detection, high-FLOPs models generally outperform low-FLOPs models, as the latter cannot fully benefit from extensive training, a phenomenon called the low-FLOPs pitfall. The traditional C2f module cannot address this phenomenon, and its larger parameter size results in higher computational resource consumption. Han et al. [21] suggests that parameters are more important than FLOPs for large-scale visual training and proposes DynamicConv [22] to improve the network, thereby addressing the low-FLOPs pitfall, although this may lead to an increase in parameters. Inspired by this, we propose a new module, C2f-GDConv, to mitigate the parameters increase caused by addressing the low-FLOPs pitfall.

**Figure 4:**
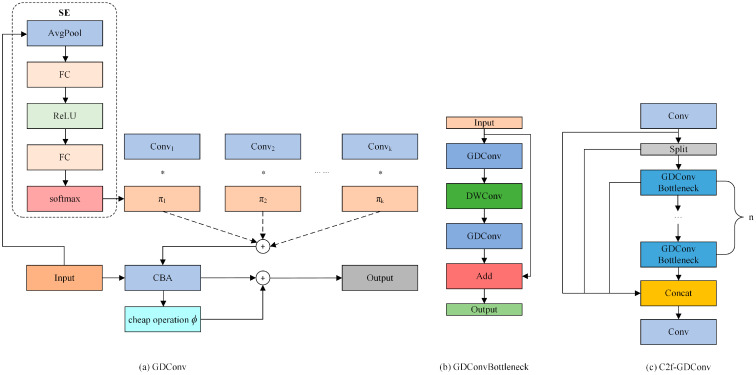
Structure of the C2f-GDConv.

We introduce GDConv by combining the GhostModule [23] with DynamicConv. GhostModule, known for its low parameters, balances the increased parameters brought by DynamicConv, while also resolving the low-FLOPs pitfall. This combination reduces the model’s parameters and computational load while maintaining the original detection accuracy. GDConv is then designed as GDConvBottleneck to replace the DarknetBottleneck in the C2f module, resulting in the C2f-GDConv module. The structure of C2f-GDConv and its related components is illustrated in Figure 4.

As shown in Figure 4a, GDConv utilizes SE attention to extract the weight π*_k_* from the input features and generates corresponding *k* convolution kernels. The output features are then generated through convolution operations and weighted fusion. By using dynamic convolutional kernels, GDConv maintains low FLOPs while increasing parameters, enabling the model to benefit during training. The output is then passed through a cheap operation *ϕ* and combined with the original output, reducing the additional parameters introduced by dynamic convolution, resulting in the final output feature map. The computation formulas for GDConv are:

(1) $$y_{d}=g(W(x)x+b(x))$$

(2) $$\begin{array}{c} W(x)=\sum_{k=1}^{K}\pi_{k}(x)W_{k},b(x)=\sum_{k=1}^{K}\pi_{k}(x)b_{k}\\ 0\leq \pi_{k}\leq 1,\sum_{k=1}^{K}\pi_{k}(x)=1 \end{array}$$

(3) $$y=Wy_{d}+\phi_{i,j}(y_{i}),\forall i=1,\ldots ,m,j=1,\ldots ,s$$

In the above formulas, x denotes the input features, y_d_ denotes the output of the dynamic convolution, g denotes the activation function, W denotes the weight matrix, and b denotes the bias vector. The attention weight is denoted by π_k_. The output of GDConv is represented by y, where y_i_ denotes to the i-th feature within y_d_, *ϕ*_i,j_ represents the j-th cheap operation used to generate the j-th ghost feature map.

### 3.3. P6 Large-Object Detection Layer

In face detection scenarios, particularly in the context of deepfake video face extraction as discussed in this paper, the low compression characteristics of deepfake videos often cause large-scale faces to be overlooked or incompletely detected on smaller feature maps. Additionally, small-scale non-face objects may be falsely detected. To address these issues, we introduce a P6 large-object detection layer [18] into the backbone network of the model. This addition increases the receptive field, enabling the capture of low-resolution, large-scale features, thereby enhancing the detection of large faces that might be missed. Specifically, we add a Conv module and a C2f-GDConv module on top of the SPPF layer in the original P5 layer. The original Conv + C2f-GDConv in the P5 layer now forms the new P5 layer, while the newly added Conv + C2f-GDConv, combined with the SPPF, constitutes the new P6 layer. The added P6 layer is highlighted in Figure 2.

### 3.4. CCFG Module

YOLOv8 utilizes the PAN-FPN feature fusion strategy, which effectively enhances the model’s feature representation through multi-level feature fusion. However, the repeated convolution and sampling operations within this structure make the model complex and increase computational costs. In the context of face detection, both the multi-scale feature learning and the efficient computational resources used are crucial. To address this issue, we propose the CCFG, as illustrated in the Neck section of Figure 2.

This module is based on the traditional CNN-based Cross-scale Feature Fusion (CCFF) [20]. However, the original CCFF structure was only designed for three detection layers and utilized the RepBlock in the Fusion module, which is incompatible with the C2f module in YOLOv8. Therefore, we optimized this structure by replacing the Fusion module with the C2f-GDConv and Concat modules, incorporating the P6 layer into the feature fusion process. By inserting multiple Conv layers in the fusion path, the module integrates features across different scales into a new, unified feature. Compared to the PAN-FPN, this design allows for the network to comprehensively learn features from various detection layers without increasing the network’s parameters and computational load, thereby avoiding the computational overhead associated with the layer-by-layer sampling approach used in PAN-FPN.

### 3.5. GSCD Head

The original detection head of YOLOv8 utilizes a decoupled head structure, splitting the detection head into regression and classification components. This design employs parallel convolutional modules to predict the feature maps, which can enhance the network’s performance in multi-class detection. However, for single-class detection tasks like face detection, this fully parallel structure can lead to unnecessary computational resource usage and offers minimal improvement in detection accuracy.

**Figure 5:**
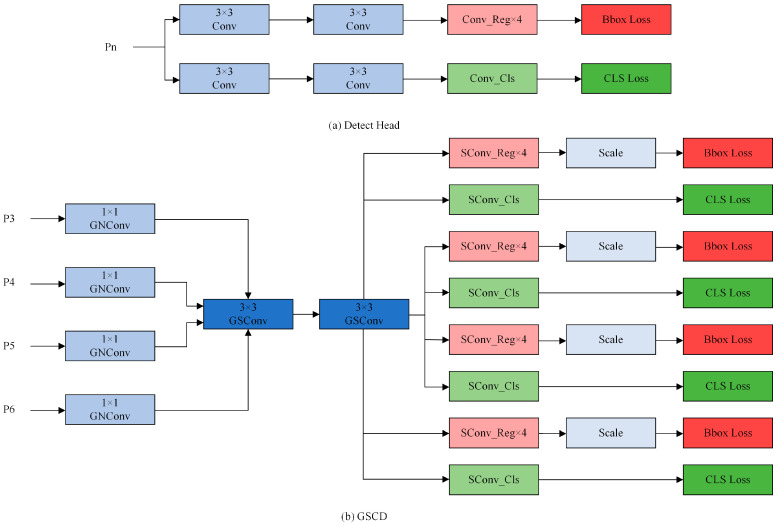
Structure of the Detect head and the GSCD.

To address these issues, we integrate Group Normalization (GN) with Shared Convolution (SConv) and introduce a novel structure named GSConv to improve the detection head. Group Normalization’s enhancement of localization and classification performance has been validated in the literature [24], while Shared Convolution reduces the model’s parameters and computational cost, thereby conserving the network’s computational resources. Compared to the original detection head, this modification not only enhances the detection head’s performance, but also significantly reduces its parameters, making the model more lightweight. Furthermore, to accommodate the addition of the P6 detection layer, the original network’s three-head detection is expanded to four-head detection. This improved detection head is termed GSCD. The structural differences between the original detection head and GSCD are illustrated in Figure 5.

The proposed GSCD head replaces the two parallel convolutions in each detection head with GSConv, and the convolutions for regression and classification are replaced with SConv. For clarity, Figure 5b depicts the four-head versions of these two types of convolutions. Each detection head uses an independent Scale layer to adjust the feature map of the regression layer, ensuring adaptability to different input scales. Additionally, a GNConv layer is added to each layer to extract features, compensating for the potential performance loss caused by using shared convolutions. Subsequent experiments demonstrate that the GSCD detection head performs well in single-class tasks, such as face detection, while effectively reducing the model’s parameters and computational load.

## 4. Experiments and Results Analysis

### 4.1. Experimental Environment and Training Parameter Configuration

Table 1 provides the training parameter settings and environment configurations used in the experiments.

### 4.2. Dataset Introduction and Improvements

#### 4.2.1. Dataset Introduction

WiderFace [25], released by the Chinese University of Hong Kong, is a widely used face detection dataset. It contains 32,203 images with a total of 393,703 annotated faces, covering 61 event categories. The dataset is characterized by its diversity and high complexity. It is divided into training, validation, and test sets (4:1:5 ratio), and is categorized into three levels of detection difficulty: Easy, Medium, and Hard.

Celeb-DF-v2 [26] is a large-scale deepfake video dataset proposed by Li et al. It contains 5639 high-quality deepfake videos of celebrities and 590 real videos sourced from YouTube. The average length of each video is 13 s, with a standard frame rate of 30 frames per second. The fake videos were generated by swapping the faces of 59 celebrities.

FaceForensics++ [27] (FF++) is a public deepfake video dataset introduced by Rössler et al. It includes 1000 real face videos and their corresponding forged videos created using four different methods: DeepFakes, Face2Face, FaceSwap, and NeuralTextures. To simulate video compression during transmission, the videos were compressed using H.264 encoding, resulting in three versions: Raw (c0), HQ (c23), and LQ (c40), representing uncompressed, high-quality low-compression, and low-quality high-compression videos, respectively. In this paper, we use the DeepFakes and FaceSwap videos that involve face replacement, with the compression version set to HQ (c23).

We utilize the WiderFace dataset for training and its test set, along with two deepfake datasets, Celeb-DF-v2 and FaceForensics++, for evaluation.

#### 4.2.2. Dataset Improvements

The WiderFace dataset contains a large number of face annotations, some of which correspond to targets with very small pixel sizes, making them difficult to detect, and thus can be disregarded in practical scenarios. Additionally, these small targets can introduce noise into the training process. To address this, we optimize the dataset.

First, we trained the original YOLOv8 network and used the resulting model weights to predict the training set of the dataset, saving the generated labels. By comparing the original training set labels with the predicted labels, we filtered out labels in the original set that had a pixel size smaller than 6 and a confidence level lower than 0.5. This resulted in an improved dataset, which we validated in subsequent experiments, demonstrating good performance.

### 4.3. Evaluation Metrics

We use Precision (P), Recall (R), Average Precision (AP), Parameters, and FLOPs as evaluation metrics. The formulas for calculating P, R, and AP are:

(4) $$P=\frac{TP}{\left(TP+FP\right)}$$

(5) $$R=\frac{TP}{\left(TP+FN\right)}$$

(6) $$AP=\int_{0}^{1}P(R)dR$$

In these formulas, TP (True Positive) represents the number of correctly identified faces, FP (False Positive) represents the number of incorrectly predicted faces, and FN (False Negative) represents the number of missed faces. P(R) denotes the precision when the recall is R.

The Parameters and FLOPs are metrics used to measure the size of a network model. Smaller values for parameters and FLOPs indicate a more compact and lightweight model, requiring fewer computational resources and placing lower demands on hardware.

### 4.4. Experimental Process and Results Analysis

#### 4.4.1. Dataset Improvement Experiment

We apply different preprocessing strategies to the WiderFace training set, using the performance of YOLOv8 on the original dataset as the baseline. Various minimum pixel thresholds are set for a comparative experiment on the dataset. The results of these experiments are presented in Table 2, with the best results highlighted in bold.

As shown in Table 2, removing labels below 8-pixel increases the AP for the Easy and Medium difficulty levels by 0.9% and 0.7%, respectively, compared to the baseline network, while the AP for the Hard difficulty level decreases by 2.1%. Removing labels below 7-pixel increases the AP for the Easy and Medium difficulty levels by 0.7% and 0.4%, respectively, but the AP for the Hard difficulty level decreases by 0.9%. Removing labels below 6-pixel results in the most balanced improvement, with the AP increasing by 0.5%, 0.3%, and 0.7% for the Easy, Medium, and Hard difficulty levels, respectively. However, after removing labels below 5-pixel, the AP for the Hard difficulty level significantly improves by 1.2%, but the AP for the Easy and Medium difficulty levels both decrease by 0.4%. Therefore, this study adopts the strategy of removing labels below 6-pixel for subsequent experiments.

#### 4.4.2. Module Comparison Experiments

We design module comparison experiments to validate the superiority of the proposed method by comparing the performance of HGStem, C2f-GDConv, CCFG, and GSCD with other innovative modules of the same type on the WiderFace test set. The experimental results are presented in Table 3, with the best results highlighted in bold, and the same type of innovative modules are separated by black lines.

In Table 3, ADown and V7Down are the downsampling methods from YOLOv9 and YOLOv7, respectively. As shown in the results, HGStem outperforms the other downsampling methods across all metrics. The AP of C2f-GDConv across all three difficulty levels of the dataset outperform other lightweight C2f modules, while having the smallest parameters. Its FLOPs are only higher than those of RepNCSPELAN4. The CCFG module has a smaller parameters and FLOPs compared to BiFPN, while achieving higher AP on the Medium and Hard difficulty levels. On the Easy level, the AP is only 0.1% lower than that of BiFPN. The GSCD demonstrates superior performance compared to the other two innovative detection heads, outperforming them in all metrics. These experimental results indicate that the proposed innovative modules offer superiority over other innovative modules.

#### 4.4.3. Ablation Experiments

We design ablation experiments to validate the effectiveness of the proposed improvements. Using YOLOv8 as the baseline network, we compare the performance of the model on the WiderFace test set by adding the proposed improvements individually and incrementally. Table 4 presents the results of the ablation experiments, where a checkmark (√) indicates the use of a specific improvement, and bold numbers represent the best results.

From Table 4, we see that replacing the dual-convolution downsampling with HGStem increases the AP by 0.5%, 0.8%, and 1.3% across the three difficulty levels, while reducing the parameters by 0.01 M and the FLOPs by 0.2 G. This further verifies the effectiveness of Stem-like modules in early feature extraction. After substituting the C2f module with the C2f-GDConv module, the model’s performance slightly decreases, with the AP dropping by 0.5% and 0.7% for the Easy and Hard difficulty levels, respectively. However, the parameters and FLOPs decrease significantly, by 0.83 M and 2.3 G, confirming the lightweight effect of this module. Adding the P6 large-object detection layer increases the AP by 0.4%, 0.4%, and 0.5% across the three difficulty levels, with the parameters increasing by 1.77 M and no change in the FLOPs, validating the benefits of the P6 layer for large-scale features. Introducing the CCFG module in the Neck section decreases the AP by 0.4% and 0.2% for the Easy and Medium difficulty levels, while increasing the AP by 1.2% for the Hard difficulty level. The parameters and FLOPs decrease by 0.65 M and 1.5 G, respectively, proving that this structure effectively integrates multi-scale features while reducing computational complexity. Implementing the GSCD detection head increases the AP by 0.5%, 0.3%, and 0.6% across the three difficulty levels, while reducing the parameters and FLOPs by 0.84 M and 1.6 G, respectively, indicating its effectiveness and lightweight nature for single-class targets such as faces. As more improvement strategies are gradually added to the network, the model’s performance improves, and the parameters and FLOPs gradually decrease.

After integrating the five proposed improvement strategies, the model achieves an AP of 0.942, 0.927, and 0.812 across the three difficulty levels, with the parameters and FLOPs of only 1.68 M and 3.5 G. Compared to the original model, the AP increased by 0.6%, 1%, and 3%. The results of the ablation experiments demonstrate that the five proposed improvement strategies enhance the YOLOv8 network to varying degrees, showing good results in both performance and lightweight design.

#### 4.4.4. Comparison Experiments

We design comparative experiments to further validate the superiority of the proposed GCS-YOLOv8 face extractor. We compare the performance of GCS-YOLOv8 with existing state-of-the-art algorithms on the WiderFace test set. The results of these comparative experiments are presented in Table 5, with the best performance highlighted in bold.

From Table 5, it can be seen that the proposed face extractor GCS-YOLOv8 achieves AP values of 0.942, 0.927, and 0.812 across the three difficulty levels, with the parameters of 1.68 MB and the FLOPs of 3.5 G. Compared to the baseline model YOLOv8, the AP values increase by 1.1%, 1.3%, and 3.7%, respectively, while the parameters and FLOPs decrease by 44.2% and 56.8%. When compared to similar face extractors YOLO5Face [18] and YOLOv8Face [35], GCS-YOLOv8 only lags behind YOLOv8Face by 0.3% in the Easy difficulty level, but outperforms both algorithms in the other difficulty levels, with the smallest parameters and slightly higher FLOPs than YOLO5Face. In comparison with advanced algorithms like SCRFD-2.5GF [17], RetinaFace [36], and LFFD [37], GCS-YOLOv8 falls short of RetinaFace by 0.5% in the Easy difficulty level, but exceeds the other two in AP values across the remaining levels. Its parameters are just 1.01 MB higher than the ultra-lightweight SCRFD-2.5GF and lower than those of the other algorithms, with the lowest FLOPs among the advanced algorithms. These comparative experiments demonstrate that the proposed GCS-YOLOv8 achieves the high AP while significantly reducing the parameters and FLOPs, ensuring that the model delivers superior performance with lower computational resource consumption.

#### 4.4.5. Visualization Result Analysis

**Figure 6:**
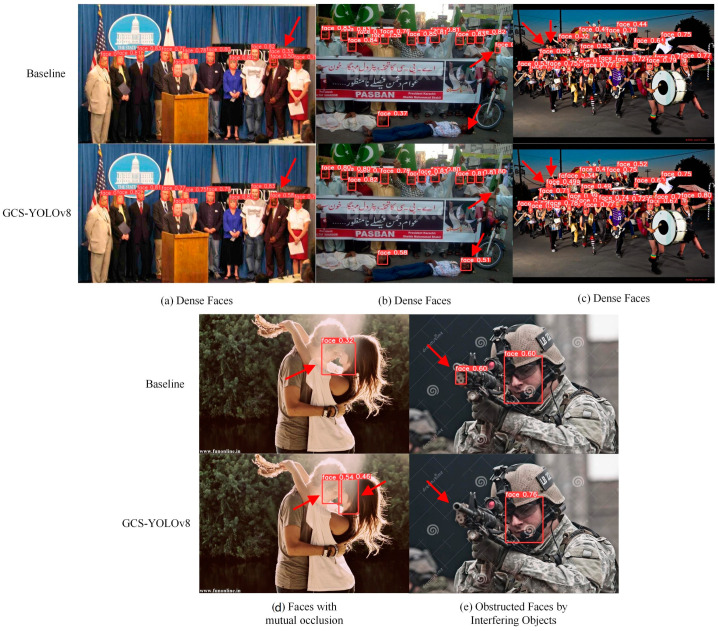
Comparison of detection effects on WiderFace test sets.

To provide a more intuitive demonstration of the effectiveness of the proposed model, we evaluate the GCS-YOLOv8 face detector on the test set of the WiderFace dataset. Visual detection samples from the WiderFace test set are shown in Figure 6, with arrows marking instances of missed or incorrect detections.

As shown in the figure, the GCS-YOLOv8 face detector performs exceptionally well in dense face scenarios, avoiding both missed detections and false positives. In contrast, the baseline YOLOv8 exhibits issues in several instances. In sample (a), YOLOv8 incorrectly detects one of the faces twice, in sample (b), it fails to detect a face on the ground while mistakenly classifying a human hand as a face, and in sample (c), it misses two faces. In the scenario of faces with mutual occlusion, YOLOv8 identifies two occluded faces in sample (d) as one, whereas GCS-YOLOv8 successfully detects both faces. When dealing with obstructed faces by interfering objects, GCS-YOLOv8 also performs robustly, whereas YOLOv8 erroneously identifies the gun in sample (e) as a face. These detection samples demonstrate that the GCS-YOLOv8 face detector is highly adaptable to various detection scenarios, consistently delivering superior performance.

**Figure 7:**
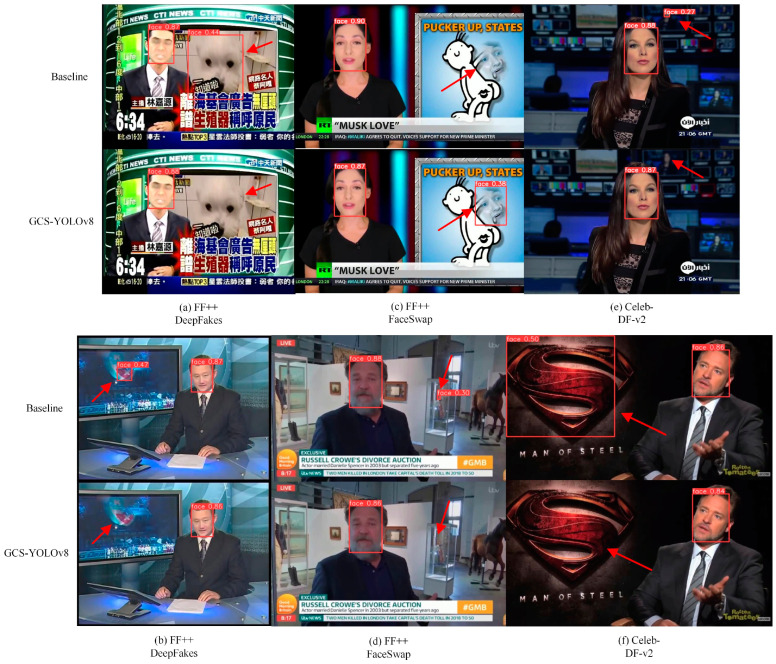
Comparison of detection effects on Celeb-DF-v2 and FF++.

To further validate the performance of the GCS-YOLOv8 face detector in the practical application scenarios proposed in this paper, we test it on the deepfake video datasets Celeb-DF-v2 and FF++. Sample detections from both datasets are shown in Figure 7.

From the figures, it can be observed that YOLOv8 misidentifies a dog as a face in sample (a) and incorrectly recognizes an unrelated object on the television as a face in sample (b). Additionally, in sample (c), it fails to detect a face generated by forgery techniques, and in sample (d), it produces a false positive. These issues may hinder subsequent forgery detection efforts. In contrast, GCS-YOLOv8 does not exhibit these errors. In sample (e), YOLOv8 detects a blurry face at a distance, but such small, blurry faces are negligible in practical face detection and deepfake detection scenarios. Furthermore, in sample (f), YOLOv8 mistakenly identifies a Superman logo as a face. GCS-YOLOv8 avoids these issues, and its detection results further support the effectiveness of the proposed dataset improvement strategy.

## 5. Conclusions

In this paper, we propose a lightweight deepfake video face extractor named GCS-YOLOv8 to assist deepfake detection, which addresses issues such as face feature blurring and misdetection caused by high compression rates in deepfake videos, as well as the high computational resources required by existing face extractors. This face extractor replaces double convolutions with HGStem for initial downsampling, capturing multi-scale features early in training and preventing false positives on small non-face objects. It introduces GDConv by combining GhostModule and DynamicConv, and designs GDConvBottleNeck to improve the C2f module, resulting in the C2f-GDConv module. This approach ensures minimal performance loss while making the model lightweight and avoiding the low-FLOPs pitfall. Adding a large target detection layer, P6, to the YOLOv8 network introduces low-resolution scale features with a larger receptive field, enabling better extraction of large-scale face features and enhancing the model’s performance. The introduction of the CCFG structure in the Neck section significantly reduces the network parameters and FLOPs while improving performance through cross-scale feature fusion. Improvements to the detection head using GN and SConv enhance the network’s capability for single-target detection and make the model more lightweight. Experimental results demonstrate that the proposed improvements achieve an AP of 0.942, 0.927, and 0.812 on the three difficulty levels of WiderFace, with parameters and FLOPs of only 1.68 M and 3.5 G, respectively, outperforming existing advanced algorithms and effectively extracting faces from deepfake videos. Future work will involve pruning and distilling the model to further reduce computational load, improve detection accuracy, and integrate the model with forgery detection systems.

## Figures and Tables

**Table 1:** Experimental parameters and environment.

Training parameters	Optimizer	SGD
	Learning rate	0.01
	Momentum	0.937
	Weight decay	0.005
	Loss function	CIoU
	Batch size	8
	Epoch	300
Experimental environment	Operation system	Windows 11
	CPU	i5-12600KF
	GPU	NVIDIA GeForce RTX 4070
	Memory size	32 GB DDR4
	Programming language	Python 3.8.0
	Module platform	Pytorch 2.0.1 + cuda 11.7

**Table 2:** Comparison of experimental results for different pixel filtering.

Models	Easy Val AP	Medium Val AP	Hard Val AP
Baseline	0.931	0.914	0.775
Remove labels below 8-pixel	**0.94**	**0.921**	0.754
Remove labels below 7-pixel	0.938	0.918	0.766
Remove labels below 6-pixel	0.936	0.917	0.782
Remove labels below 5-pixel	0.927	0.91	**0.787**

Bolded data indicates the best data.

**Table 3:** Module comparison experiment results.

Models	Easy Val AP	Medium Val AP	Hard Val AP	Parameters/MB	FLOPs
Baseline	0.936	0.917	0.782	3.01	8.1 G
YOLOv8+HGStem	**0.941**	**0.925**	**0.795**	**3.00**	**7.9 G**
YOLOv8+Adown [28]	0.935	0.911	0.772	3.01	8.0 G
YOLOv8+V7Down [29]	0.932	0.914	0.775	3.01	8.0 G
YOLOv8+C2f-GDConv	**0.931**	**0.917**	**0.775**	**2.18**	7.9 G
YOLOv8+C2f-SCConv [30]	0.927	0.911	0.762	2.86	7.9 G
YOLOv8+RepNCSPELAN4 [28]	0.914	0.902	0.757	2.19	**6.1 G**
YOLOv8+C2f-DualConv [31]	0.929	0.915	0.774	2.85	8.0 G
YOLOv8+CCFG	0.932	**0.915**	**0.794**	**1.96**	**6.6 G**
YOLOv8+BiFPN [32]	**0.933**	0.911	0.793	2.78	8.1 G
YOLOv8+GSCD	**0.939**	**0.92**	**0.788**	**2.36**	**6.5 G**
YOLOv8+RFAHead [33]	0.933	0.91	0.779	3.93	8.4 G
YOLOv8+DBBHead [34]	0.93	0.919	0.785	5.91	11.7 G

Bolded data indicates the best data.

**Table 4:** Ablation experiment results.

Models						Easy Val AP	Medium Val AP	Hard Val AP	Parameters/MB	FLOPs
Baseline	HGStem	C2f-GDConv	P6	CCFG	GSCD					
√						0.936	0.917	0.782	3.01	8.1 G
√	√					0.941	0.925	0.795	3.00	7.9 G
√		√				0.931	0.917	0.775	2.18	5.8 G
√			√			0.94	0.921	0.787	4.78	8.1 G
√				√		0.932	0.915	0.794	1.96	6.6 G
√					√	0.939	0.92	0.788	2.36	6.5 G
√	√	√				0.937	0.921	0.789	2.17	5.6 G
√	√	√	√			0.939	0.925	0.793	3.61	5.8 G
√	√	√	√	√		0.938	0.925	0.803	2.20	4.6 G
√	√	√	√	√	√	**0.942**	**0.927**	**0.812**	**1.68**	**3.5 G**

Bolded data indicates the best data.

**Table 5:** Comparison experiment results.

Models	Easy Val AP	Medium Val AP	Hard Val AP	Parameters/MB	FLOPs
Baseline	0.931	0.914	0.775	3.01	8.1 G
YOLO5Face	0.936	0.915	0.805	1.73	**2.1 G**
YOLOv8Face	0.945	0.922	0.79	3.08	8.3 G
SCRFD-2.5GF	0.937	0.921	0.778	**0.67**	2.5 G
RetinaFace	**0.949**	0.919	0.768	29.50	37.6 G
LFFD	0.910	0.881	0.780	2.15	9.25 G
GCS-YOLOv8 (Ours)	0.942	**0.927**	**0.812**	1.68	3.5 G

Bolded data indicates the best data.

## Data Availability

The original contributions presented in the study are included in the article, further inquiries can be directed to the corresponding authors.
